# Resolution of Right Atrial Pacemaker Lead Thrombosis After Rivaroxaban Failure: A Case Report

**DOI:** 10.1155/cric/6121153

**Published:** 2026-09-21

**Authors:** Bowen Wang, Zhan Zhang, Qi Guo, Yanling Li, Ping Xie

**Affiliations:** ^1^ Department of Cardiovascular Medicine, Gansu Provincial Hospital, Lanzhou City, Gansu Province, China, gsyy.cn; ^2^ Anzhen Hospital, Capital Medical University, Beijing, China, ccmu.edu.cn

**Keywords:** anticoagulation therapy, INR, pacemaker lead thrombosis, warfarin

## Abstract

**Background:**

Pacemaker lead thrombosis is a rare but serious complication of cardiac implantable electronic devices (CIEDs) that often mimics infection; its management, given the lack of standardized treatment strategies, requires individualized anticoagulation and dynamic imaging.

**Case Presentation:**

A 65‐year‐old woman with heart failure and diabetes mellitus had previously undergone dual‐chamber pacemaker implantation following radiofrequency catheter ablation. After the subsequent occurrence of ventricular tachycardia, the device was upgraded to a cardiac resynchronization therapy defibrillator (CRT‐D). She later experienced recurrent atrial flutter accompanied by worsening heart failure. The timing of atrial flutter recurrence was uncertain, and the patient had not received anticoagulation therapy. Preoperative transesophageal echocardiography (TEE) revealed a mobile mass measuring 10 × 3.5 mm attached to the right atrial lead. Comprehensive clinical and laboratory evaluation showed no evidence of infection. Anticoagulation with rivaroxaban at 20 mg once daily was initiated; however, no reduction in thrombus size was observed after 1 month. The treatment regimen was therefore switched to warfarin, with an initial target international normalized ratio (INR) of 2.0–3.0. Follow‐up TEE performed 3 months later demonstrated enlargement of the thrombus to 18 × 3.8 mm. The target INR was subsequently intensified to 2.5–3.0, after which the thrombus gradually regressed. Serial TEE examinations confirmed complete thrombus resolution by 6 months. No embolic or bleeding complications occurred during the treatment course.

**Conclusion:**

This case underscores the inadequacy of NOAC monotherapy in selected high‐risk patients with lead‐associated thrombus, and demonstrates that INR‐guided warfarin therapy with individualized adjustment was effective in achieving thrombus resolution.

## 1. Introduction

Cardiac implantable electronic devices (CIEDs), including pacemakers and defibrillators, have become standard in the management of arrhythmias and heart failure [1]. Although generally safe, these devices are associated with rare but potentially serious complications, such as pacemaker lead‐associated thrombosis [1]. This condition is often underdiagnosed due to its overlapping clinical features with device‐related infective endocarditis and nonspecific symptoms.

Right atrial thrombi, especially those involving pacemaker leads, are less frequently encountered than left atrial thrombi but can lead to serious complications including pulmonary embolism [2, 3]. High‐resolution imaging, particularly transesophageal echocardiography (TEE), is crucial for detecting and monitoring such thrombi, as transthoracic echocardiography (TTE) is often limited in its ability to visualize right atrial structures.

The optimal anticoagulation regimen for managing lead‐associated thrombus remains unclear. Although nonvitamin K antagonist oral anticoagulants (NOACs) are widely prescribed for atrial fibrillation (AF), their efficacy in patients with CIED‐associated thrombus is not well established. Here, we report a case of right atrial lead thrombosis unresponsive to rivaroxaban that was successfully managed with individualized, international normalized ratio (INR)–guided warfarin therapy. Serial TEE provided critical imaging guidance throughout treatment.

## 2. Case Presentation

A 65‐year‐old woman with a history of paroxysmal AF, atrial flutter, Type 2 diabetes mellitus, and heart failure with reduced ejection fraction (LVEF 33%) presented. Six years earlier, the patient had undergone radiofrequency catheter ablation combined with permanent dual‐chamber pacemaker implantation at our institution for paroxysmal AF and atrial flutter complicated by first‐degree atrioventricular block, second‐degree atrioventricular block, and complete right bundle branch block. Left bundle branch area pacing was used. Three years earlier, because of paroxysmal ventricular tachycardia, the original dual‐chamber pacemaker was upgraded at our institution to a cardiac resynchronization therapy defibrillator (CRT‐D), consisting of left bundle branch area pacing combined with a right ventricular defibrillation lead. The perioperative course was uneventful, with no apparent discomfort or complications, and the patient recovered well and was discharged in stable condition.

**Figure 1:**
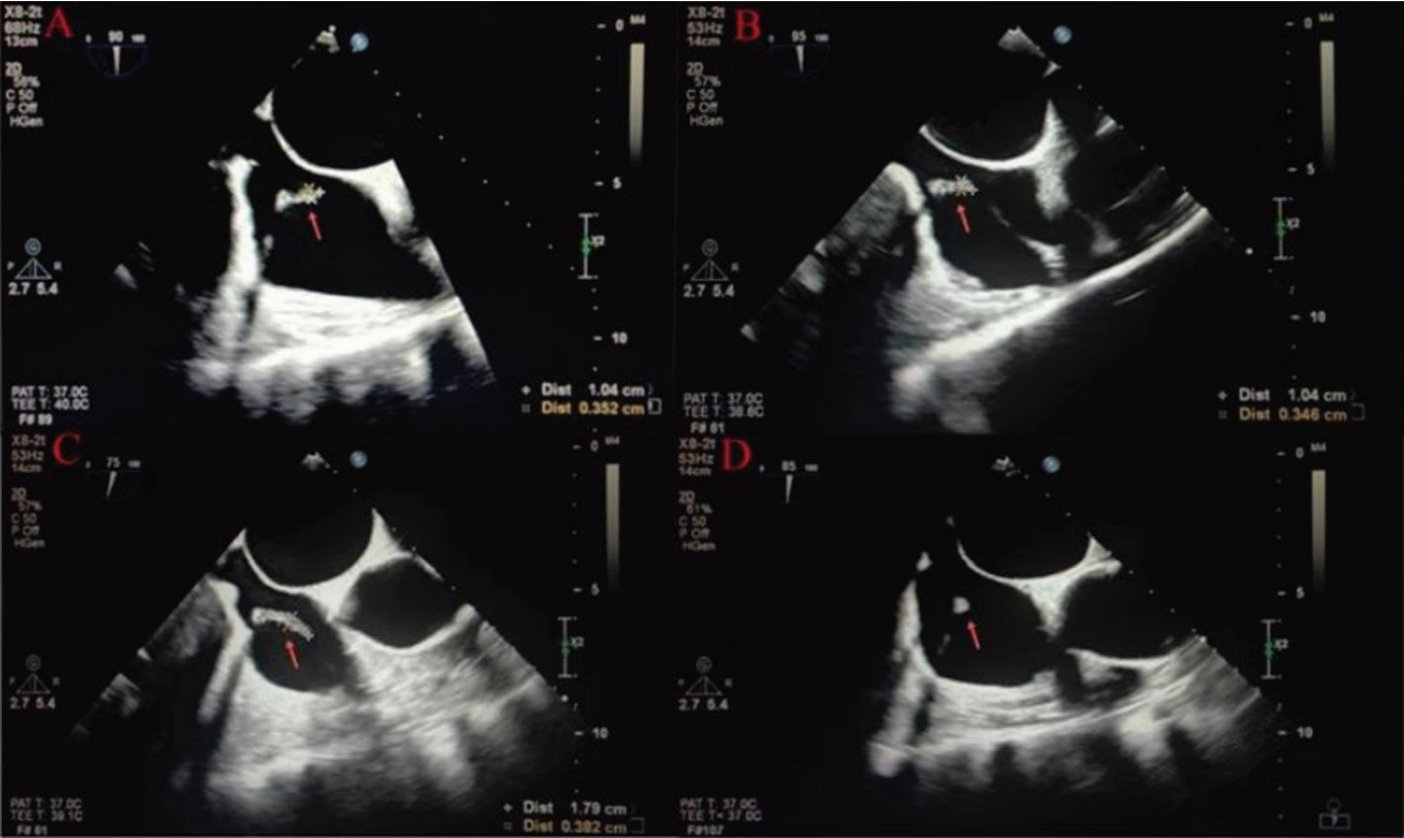
Serial transesophageal echocardiography (TEE) images of the right atrial lead thrombus. (A) Initial TEE image prior to admission showing a 10 × 3.5 mm mobile, hyperechoic mass attached to the right atrial lead, suggestive of thrombus. (B) One month after initiation of rivaroxaban therapy, the thrombus remained unchanged in morphology and size. (C) Three months after switching to warfarin (INR 2.0–3.0), the thrombus had enlarged to 18 × 3.8 mm, indicating inadequate anticoagulant effect. (D) Six months after INR was adjusted to 2.5–3.0, complete resolution of the thrombus was achieved.

One week before the current admission, the patient developed chest tightness, exertional dyspnea, and palpitations. Outpatient electrocardiographic monitoring confirmed recurrent atrial flutter, and a plan was made for repeat radiofrequency ablation. Preprocedural TEE (partial static TEE images of the right atrial lead thrombus are shown in the attachment under figure section) revealed a mobile hyperechoic mass measuring 10 × 3.5 mm attached to the right atrial lead, raising suspicion for thrombus (Figure 1A).

**Figure 2:**
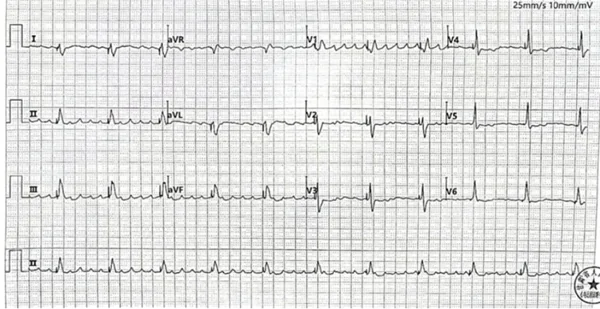
Electrocardiogram on admission. Atrial flutter with ventricular‐paced rhythm (VVI mode), consistent with pacemaker‐dependent conduction.

The patient was admitted for further evaluation and management. On admission, her vital signs were stable: temperature 36.3°C, heart rate 72 bpm, respiratory rate 18 breaths/min, blood pressure 125/72 mmHg, and BMI 28.5 kg/m^2^. Cardiac examination was unremarkable. There was no redness, swelling, or tenderness at the left subclavian device site. The electrocardiogram showed atrial flutter with a ventricular‐paced rhythm (VVI mode) (Figure 2). TTE did not reveal any abnormal echogenicity on the right atrial lead.

**Figure 3:**
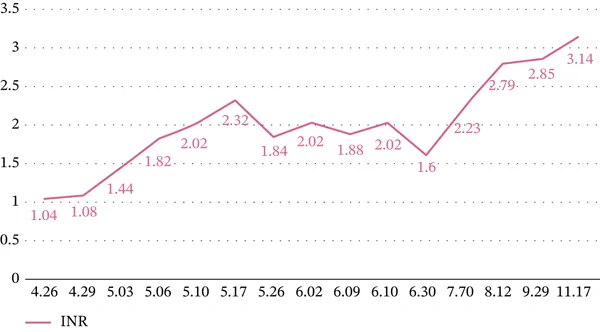
INR monitoring trend chart. INR values over time during warfarin therapy. Thrombus progression occurred at INR 2.0–2.5; resolution followed INR escalation to 2.5–3.0, as confirmed by serial TEE.

Rivaroxaban (20 mg once daily) was initiated for anticoagulation. However, 1 month later, follow‐up TEE showed that the thrombus remained unchanged in size and appearance (Figure 1B). Due to the lack of therapeutic response, rivaroxaban was discontinued, and warfarin therapy was initiated with a target INR of 2.0–3.0. During this period, the patient′s atrial flutter spontaneously resolved. At the 3‐month follow‐up, TEE revealed that the thrombus had enlarged to 18 × 3.8 mm (Figure 1C), suggesting subtherapeutic anticoagulation. Subsequently, the target INR was increased to 2.5–3.0. The warfarin dosage was adjusted accordingly, with frequent outpatient INR monitoring (Figure 3). At 6 months, repeat TEE demonstrated complete thrombus resolution (Figure 1D). No embolic or bleeding events occurred throughout the course of anticoagulation.

## 3. Discussion

Pacemaker lead thrombosis remains a rare but potentially serious complication of CIEDs. The true incidence is likely underestimated due to diagnostic challenges and overlap with other conditions, particularly lead‐related infective endocarditis. This case highlights the importance of early recognition, imaging surveillance, and individualized anticoagulation strategies in managing device‐related thrombosis.

The formation of thrombus on a pacemaker lead is multifactorial. In this case, several contributory factors were identified: (1) an unknown duration of recurrent atrial arrhythmia without anticoagulation, which increases the risk of atrial thrombus formation; (2) multiple device interventions, including initial pacemaker implantation and later upgrade to CRT‐D; (3) long‐standing endocardial lead placement; and (4) comorbid conditions such as Type 2 diabetes mellitus and heart failure, which are associated with systemic prothrombotic states. Although most atrial thrombi originate from the left atrial appendage in AF [4, 5], right atrial thrombi, though less common (reported in 0.6%–0.75%), may lead to serious complications such as pulmonary embolism or paradoxical embolism through a patent foramen ovale [2, 3].

Distinguishing thrombus from device‐related infection is essential, as management differs significantly. In this case, the absence of fever, procalcitonin and interleukin‐6 were both negative, and the complete blood count was unremarkable, and negative blood cultures argued against infective endocarditis. TTE failed to detect the mass, whereas TEE clearly showed a thrombus on the lead in the right atrium, ruling out thrombi in the left atrium or left atrial appendage and infective endocarditis. TEE is superior in evaluating lead‐associated thrombus due to its higher resolution and better visualization of atrial structures. In contrast, CT angiography may be limited by metallic artifacts from the leads [6]. TEE allowed not only initial detection but also dynamic monitoring of the thrombus over time, guiding anticoagulation adjustment.

Currently, no consensus exists regarding the optimal anticoagulation regimen for pacemaker lead thrombosis. Although NOACs such as rivaroxaban are widely used in AF management and have demonstrated efficacy comparable to warfarin in venous thromboembolism [7], their utility in CIED‐associated thrombosis is less clear. In this case, rivaroxaban failed to reduce the thrombus burden after 1 month. The patient was switched to warfarin, initially targeting an INR of 2.0–3.0. However, thrombus progression was observed, prompting adjustment of the target INR to 2.5–3.0. This change was associated with progressive thrombus shrinkage and eventual resolution. These findings suggest that standard NOAC therapy may be insufficient in cases of high thrombotic burden or intravascular foreign bodies. Warfarin remains a viable first‐line treatment in such cases, provided that INR is carefully monitored and titrated [8].

Possible mechanisms for the lack of response to rivaroxaban include (1) the dense structure of thrombus formed on the foreign surface of the lead, which may limit local penetration of NOACs; (2) high shear stress around the lead in the right atrium facilitating continuous thrombin activation; (3) prothrombotic conditions such as diabetes mellitus and heart failure, where warfarin offers more comprehensive anticoagulation through multitarget inhibition of vitamin K‐dependent factors; and (4) high BMI potentially affecting NOAC plasma concentrations. In contrast, INR‐guided warfarin allowed individualized titration to a higher target (2.5–3.0), which ultimately led to complete thrombus resolution in this case.

This case aligns with previous reports that emphasize the diagnostic and therapeutic complexities of lead‐associated thrombosis. D′Aloia et al. [9] described a large right atrial thrombus attached to a pacing lead causing pulmonary embolism. Similarly, Buttigieg et al. [10] reported a CRT patient with thrombus confirmed by TEE and histopathology, ultimately requiring surgical removal. Domain et al. [11] observed thrombus resolution with intravenous heparin in another early‐detected case [7]. Our patient demonstrated full resolution with oral warfarin alone, supporting its efficacy when appropriately monitored. The increase in thrombus size at lower INR levels (2.0–2.5) highlights the need for a higher therapeutic threshold in selected cases.

## 4. Conclusion

In conclusion, pacemaker lead thrombosis should be considered in patients with CIEDs presenting with arrhythmias or suspected embolic phenomena. Comprehensive evaluation using TEE and laboratory markers is essential for differentiating thrombus from infective endocarditis. Anticoagulation should be individualized based on thrombus characteristics, patient comorbidities, and treatment response. Warfarin, with INR targeted to 2.5–3.0, remains an effective option in patients with rivaroxaban failure or high thrombotic burden. Serial TEE provides indispensable guidance for diagnosis and therapeutic monitoring.

## Author Contributions

Bowen Wang wrote the initial draft of the manuscript. Zhan Zhang conducted a comprehensive literature review and assisted in manuscript writing. Qi Guo and Yanling Li were responsible for organizing the clinical data and acquiring the echocardiographic images. Ping Xie supervised the study and critically reviewed and revised the manuscript for important intellectual content.

## Funding

This study was supported by the Gansu Province Traditional Chinese Medicine Scientific Research Project (GZKZ‐2021‐7), Gansu Provincial Drug Scientific Research Project (2025GSMPA059), Gansu Joint Research Fund (24JRRA886), and National Natural Science Foundation of China (NSFC) Regional Cultivation Project of Gansu Provincial Hospital (25GSSYA‐10).

## Disclosure

All authors have read and approved the final version of the manuscript, Ping Xie had full access to all of the data in this study and takes complete responsibility for the integrity of the data and the accuracy of the data analysis.

## Consent

Written informed consent was obtained from the patient for publication of this case report and any accompanying images. A copy of the written consent is available for review by the editor in chief of this journal.

## Conflicts of Interest

The authors declare no conflicts of interest.

## Data Availability

Data sharing is not applicable to this article as no datasets were generated or analyzed during the current study.
